# Status of β_1_-Adrenoceptor Signal Transduction System in Cardiac Hypertrophy and Heart Failure

**DOI:** 10.31083/j.rcm2409264

**Published:** 2023-09-21

**Authors:** Naranjan S. Dhalla, Sukhwinder K. Bhullar, Adriana Adameova, Karina Oliveira Mota, Carla Maria Lins de Vasconcelos

**Affiliations:** ^1^Institute of Cardiovascular Sciences, St. Boniface Hospital Albrechtsen Research Centre, and Department of Physiology and Pathophysiology, Max Rady College of Medicine, University of Manitoba, Winnipeg, MB R2H 2A6, Canada; ^2^Department of Pharmacology and Toxicology, Faculty of Pharmacy, Comenius University and Centre of Experimental Medicine, Institute for Heart Research, Slovak Academy of Sciences, 811 03 Bratislava, Slovakia; ^3^Heart Biophysics Laboratory, Department of Physiology, Center for Biological and Health Sciences, Federal University of Sergipe, 73330 Sergipe, Brazil

**Keywords:** adaptive cardiac hypertrophy, maladaptive cardiac hypertrophy, heart failure, β_1_-adrenoceptors, intracellular Ca^2+^, adenylyl cyclase, cardiac function

## Abstract

Although β_1_-adrenoceptor (β_1_-AR) signal transduction, which maintains cardiac function, is downregulated in failing hearts, the mechanisms for such a defect in heart failure are not fully understood. Since cardiac hypertrophy is invariably associated with heart failure, it is possible that the loss of β_1_-AR mechanisms in failing heart occurs due to hypertrophic process. In this regard, we have reviewed the information from a rat model of adaptive cardiac hypertrophy and maladaptive hypertrophy at 4 and 24 weeks after inducing pressure overload as well as adaptive cardiac hypertrophy and heart failure at 4 and 24 weeks after inducing volume overload, respectively. Varying degrees of alterations in β_1_-AR density as well as isoproterenol-induced increases in cardiac function, intracellular ${\mathrm{Ca}}^{2+}$-concentration in cardiomyocytes and adenylyl cyclase activity in crude membranes have been reported under these hypertrophic conditions. Adaptive hypertrophy at 4 weeks of pressure or volume overload showed unaltered or augmented increases in the activities of different components of β_1_-AR signaling. On the other hand, maladaptive hypertrophy due to pressure overload and heart failure due to volume overload at 24 weeks revealed depressions in the activities of β_1_-AR signal transduction pathway. These observations provide evidence that β_1_-AR signal system is either unaltered or upregulated in adaptive cardiac hypertrophy and downregulated in maladaptive cardiac hypertrophy or heart failure. Furthermore, the information presented in this article supports the concept that downregulation of β_1_-AR mechanisms in heart failure or maladaptive cardiac hypertrophy is not due to hypertrophic process *per se*. It is suggested that a complex mechanism involving the autonomic imbalance may be of a critical importance in determining differential alterations in non-failing and failing hearts.

## 1. Introduction 

In Canada, more than 100,000 patients with heart failure are diagnosed annually and about 2.6 million adults aged 20 and over are living with this heart disease. Since heart failure is one of the top reasons for hospitalization, the associated healthcare costs have been estimated to reach $2.8 billion by 2030 in this country [1, 2, 3, 4]. However, it should be pointed out that significant advances have been made for the development of medical therapies, which are used for the treatment of this disease. Several interventions have reduced morbidity, mortality, and economic burden of this devastating disorder, and in fact a great deal of effort is being made to further improve its pharmacotherapy [5, 6, 7, 8, 9, 10, 11]. Although extensive research is also being done to understand the pathogenesis of heart failure, the exact mechanism for its progression remains unclear at present [12, 13, 14, 15, 16, 17, 18]. Nonetheless, it is evident that heart failure is a complex problem, which is associated with different disorders such as cardiac dysfunction, cardiac arrhythmias, loss of adrenergic support, exercise intolerance and fluid retention. Since a number of vasoactive hormones are elevated in heart failure, several hormone receptor antagonists are now available for its therapy. In this regard, guanine nucleotide protein coupled receptors (GPCRs) have been identified as the most promising targets for drug discovery and a few of their blockers have been shown to exert beneficial effects in heart failure [19, 20, 21, 22, 23, 24].

It is noteworthy that β-adrenergic receptors (β-AR) are the most prominent class of GPCRs, which along with their modulators, are shown to play a critical role in cardiac health and disease [25, 26, 27, 28, 29, 30, 31, 32, 33, 34, 35, 36, 37, 38]. Since alterations in β-AR mechanisms are reported in heart failure, these targets have been manipulated to achieve clinically relevant therapies [39, 40, 41, 42]. Furthermore, attenuated responses of the heart to sympathetic stimulation have been observed at different stages of heart failure [28, 43, 44, 45]. The activities of various components of β_1_-AR system are unaltered, upregulated, or downregulated in different types of heart failure [42, 46]. Since cardiac hypertrophy is generally associated with development of heart failure [23, 47, 48, 49, 50, 51, 52], it is not clear whether upregulation or downregulation of β_1_-AR mechanisms are involved in adaptive or maladaptive cardiac hypertrophy [53, 54, 55, 56, 57, 58]. In this article, we have briefly reviewed the role of β_1_-AR signaling activation in the regulation of cardiac function upon stimulation of the sympathetic nervous system (SNS). Furthermore, the status of this system in the development of cardiac hypertrophy and heart failure is discussed. We have also reviewed the evidence regarding β_1_-AR signal alterations in adaptive and maladaptive cardiac hypertrophy due to pressure overload. In addition, some observations regarding changes in β_1_-AR mechanisms in adaptive hypertrophy and heart failure due to volume overload are described to evaluate the role of hypertrophic process in heart failure.

## 2. Role of β-AR Signal Transduction in Cardiac Function

It is now well known that stimulation of β_1_-AR signal transduction by activation of the SNS or exogenous catecholamines for a short duration augments cardiac function and produces cardiac hypertrophy whereas its stimulation for a prolonged period results in heart failure. Furthermore, several β_1_-AR blockers have been reported to exert cardiodepressant action under physiological conditions but improve cardiac function in heart failure [27, 28, 29, 30, 31, 34, 35, 36, 37, 38, 39, 40, 41, 59, 60, 61, 62, 63]. The activation of β_1_-AR stimulates adenylyl cyclase activity to form $3^{\prime }$-$5^{\prime }$-cyclic adenosine monophosphate (cyclic AMP) in the myocardium. The elevated level of cyclic AMP promotes protein kinase A (PKA)-mediated phosphorylations of different ${\mathrm{Ca}}^{2+}$-handling proteins in the sarcolemma and sarcoplasmic reticulum for increasing the intracellular concentration of ${\mathrm{Ca}}^{2+}$ ([${\mathrm{Ca}}^{2+}$]_i_) and producing positive inotropic effect in the heart [39, 64, 65, 66, 67, 68, 69]. The increased activation of β_1_-AR signal transduction is considered to provide circulatory support during early stages of heart failure [70, 71, 72, 73] but prolonged stimulation triggers β_1_-AR desensitization in the failing heart [42, 55, 69, 74, 75, 76, 77, 78, 79, 80]. Such changes due to elevated levels of circulatory catecholamines or prolonged stimulation of β_1_-AR system are associated with worsening cardiac outcome, cardiac dysfunction and sudden cardiac death [41, 63, 81, 82, 83, 84, 85, 86].

**Fig. 1.**
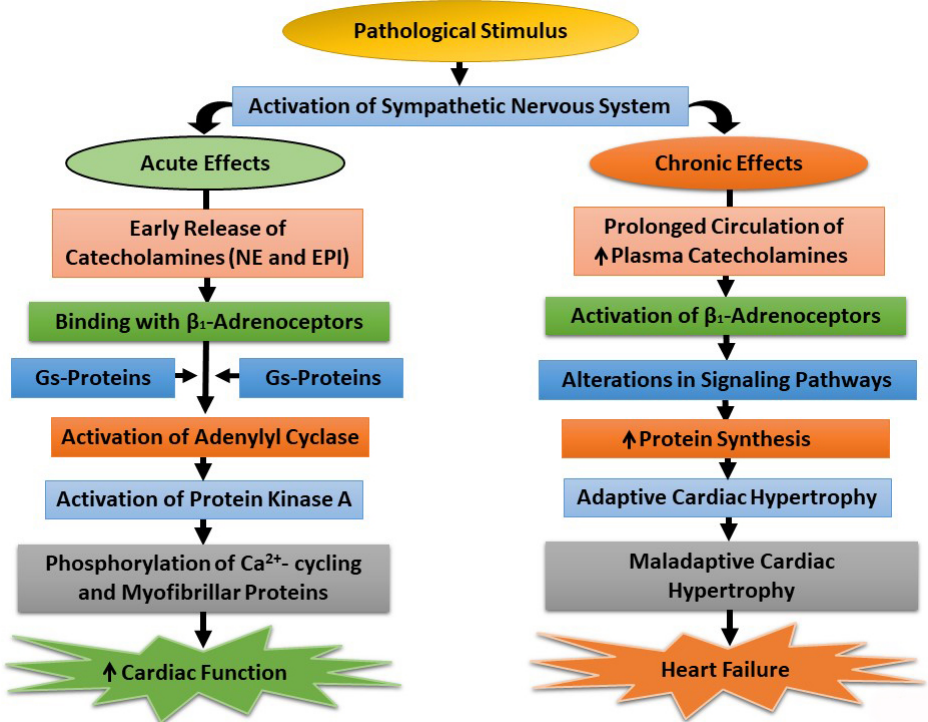
**Acute and chronic effects of the sympathetic nervous system on β-adrenoceptor-mediated signal transduction components**. NE, norepinephrine; EPI, epinephrine; Gs-Proteins, stimulatory guanine nucleotide proteins; ↑, increased.

It is pointed out that the β-AR family in healthy human heart comprises subtypes that include 80% β_1_-AR, 20% β_2_-AR and about 3% β_3_-AR [22, 87, 88, 89]. β_1_-AR subtype displays localization in the sarcolemma in the heart whereas β_2_-AR and β_3_-AR subtypes are mainly confined to the T-tubular network [90, 91]. The density of β_1_-ARs is reduced by about 50% depending upon the severity of heart failure, whereas the β_2_-AR density remains unchanged. A substantial reduction in β_1_-AR receptor density in heart failure has been shown to be due to downregulation of these receptors [44, 71, 72, 92, 93]. It should be mentioned that the activation of both β_1_-AR and β_2_-AR subtypes occurs with different potencies by catecholamines (norepinephrine and epinephrine) in general. β_1_-ARs are coupled to Gα_s_-proteins and β_2_-ARs are coupled to both Gα_s_-and Gα_i_-proteins. The acute activation of β_1_-AR through Gα_s_-proteins produces positive chronotropic and inotropic responses as well as cardiac hypertrophy whereas the chronic stimulation of β_1_-AR is associated with heart failure. The effects of both acute and chronic stimulation of the SNS are illustrated in Fig. 1. It needs to be emphasized that acute stimulation of β_1_-AR system results in adaptive hypertrophy whereas prolonged β_1_-AR signaling accounts for the development of maladaptive hypertrophy and subsequent heart failure [53, 94, 95, 96, 97]. Furthermore, overexpression of β_1_-AR in transgenic mice has also been reported to exhibit depressed cardiac function, progressive hypertrophy, and myocardial fibrosis [54, 98]. On the other hand, Gα_i_-protein mediated signaling via β_2_-AR is generally believed to be cardioprotective due to its anti-apoptotic and anti-fibrotic effects [99, 100].

In certain types of heart failure such as that due to aortic stenosis, it has been reported that β_2_-AR signaling may change to β_1_-AR-like signaling, become more susceptible to ischemic injury and contribute to the development of heart failure [101, 102]. It has been suggested that such pathological manifestations of β_2_-AR overexpression are mediated primarily by Gα_s_- proteins rather than Gα_i_-proteins [102]. Thus, it has been indicated that β_2_-AR signaling may be either protective or deleterious in the heart depending on transducer coupling with G-proteins [103, 104, 105, 106, 107, 108]. It should also be noted that both β_1_-AR and β_2_-AR subtypes are coupled to β-arrestins, which may induce cardioprotective signaling cascades in the heart. Although the role of β_3_-AR in cardiac pathology is unclear, some studies have suggested that β_3_-AR may be involved in the development of heart disease [89, 109, 110, 111, 112]. The β_3_-AR expression in the myocardium has been shown to be upregulated in heart failure [67, 113, 114]. In addition, β_3_-AR has been reported to signal through endothelial nitric oxide synthase/nitric oxide/cyclic guanosine monophosphate (eNOS/NO/cGMP) pathway for the attenuation of cardiac contractility [90]. While extensive work needs to be carried out for establishing the exact role of both β_2_-AR and β_3_-AR signaling systems in cardiac hypertrophy and heart failure, there is overwhelming evidence that β_1_-AR signal transduction is activated. In this regard, it is noteworthy that blocking β_1_-AR signaling by several antagonists such as carvedilol, metoprolol, atenolol, and bisoprolol has been shown cardioprotection and other beneficial effects in heart failure [73, 108, 115, 116, 117, 118, 119, 120, 121, 122, 123, 124, 125, 126, 127, 128, 129].

## 3. Role of β_1_-AR Signal Transduction in Cardiac Hypertrophy and Heart Failure

Several studies have indicated that a wide variety of both extrinsic and intrinsic stimuli induce activation of different signal transduction pathways to increase the muscle mass for the occurrence of cardiac hypertrophy. This process is initiated by mechanical stress as well as different hormones, cytokines and growth factors that are sensed by different receptors in the cell membrane of cardiomyocytes. It is evident that cardiac hypertrophy at initial stages is an adaptive process in which the heart does not show any structural abnormalities and cardiac function is usually unaltered or augmented [25, 56, 130, 131, 132, 133, 134]. However, if the stimulus is not removed within a certain time period, there occurs a transition of adaptive hypertrophy to maladaptive hypertrophy, which exhibit a set of complexities, including cardiac remodeling, cardiac dysfunction, metabolic alterations, electrophysiological defects and increased ventricular wall stress. Progressive metabolic alterations in maladaptive hypertrophy are considered to result in the progression of subcellular abnormalities for ${\mathrm{Ca}}^{2+}$-handling, cardiac dysfunction and heart failure [51, 57, 58]. The loss of inotropic mechanism in the hypertrophied heart has been reported to occur due to changes in membrane receptors, protein kinase activities, and associated signal transduction system as well as defects in subcellular organelles during the progression of heart failure [23, 34, 49, 50, 52, 69, 135, 136, 137, 138, 139, 140, 141, 142, 143].

Involvement of β_1_-AR signaling in both adaptive and maladaptive hypertrophy as well as in heart failure is now well established [30, 37, 42, 54, 140] and the SNS is considered to regulate the status of β_1_-AR signal pathway during occurrence of these phenotypes. At early stages, activation of the SNS and subsequent elevation in the levels of plasma norepinephrine and epinephrine stimulate β_1_-AR and increase cardiac contractile force. However, prolonged hyperactivity of the SNS and elevated plasma catecholamines result in the derangement of one or more components of the β_1_-AR signaling transduction system, including β_1_-AR, Gs-proteins, adenylyl cyclase, β_1_-AR-Gs-protein coupling, and Gs-protein-adenylyl cyclase interactions. It is pointed out that an increase in Gs-protein or content results in augmenting cardiac function by increasing the adenylyl cyclase activity whereas an increase in $G_{\text{i}}$-protein activity or content is known to depress cardiac function by decreasing the adenylyl cyclase activity. Furthermore, exposure of cardiomyocytes to high amount of norepinephrine has been shown to cause a reduction in β_1_-AR expression, adenylyl cyclase activity, and contractile activity. Thus, excessive circulating levels of catecholamines can be seen to induce abnormalities in the β_1_-AR signal transduction pathway and result in cardiac dysfunction [133, 135, 144, 145, 146, 147, 148].

Depressed sensitivity of β_1_-AR to catecholamines as well as reduction in β_1_-AR number are reported to occur in heart failure [149]. Furthermore, overexpression of β_1_-AR in the heart in transgenic mice was found to develop hypertrophy at young age followed by progressive heart failure in later life [54, 98, 150, 151, 152]. Chronic stimulation of β_1_-AR by agonists such as isoproterenol has also been observed to induce cardiac hypertrophy [53] due to activation of PKA by elevated levels of cyclic AMP. Another study has indicated that β_1_-AR signaling stimulates hypertrophy in a PKA-independent manner via the activation of cyclic AMP binding protein, Epac [153]. However, other investigators have shown that mice overexpressing PKA are protected against isoproterenol-induced cardiac hypertrophy [154]. It is also pointed that the level of Gi-proteins is elevated in heart failure and this reduces cyclic AMP content for overall depression in β_1_-AR-mediated signaling [68]. Since PKA signaling microdomains regulate ${\mathrm{Ca}}^{2+}$-handling, it has been suggested that some PKA catalytic subunit may cause maladaptive hypertrophy and result in heart failure [48]. It should also be mentioned that PKA may directly enhance the stimulation of calcium-calmodulin kinase (CaMKII) or calcineurin/nuclear factor of activated T cells (NFAT) signaling [155]. Furthermore, the activation of PKA has also been suggested to inhibit cardiac hypertrophy via some signaling protein changes such as histone deacetylases (HDAC)5 phosphorylation or HDAC4 proteolysis [156]. While most of these observations support the view that β_1_-AR stimulation results in cardiac hypertrophy and progression to heart failure [53, 93, 94, 118, 125, 157], the specific mechanisms remain unclear because of the complex nature of β_1_-AR signaling transduction pathway. It is also likely that changes in β_1_-AR signaling may depend on the stage and type of hypertrophy and heart failure.

## 4. Dependence of Changes in β_1_-AR Signal Transduction on Type and Stage of Pathological Stimulus

Since hypertrophy and heart failure are known to occur in response to several pathological stimuli, it was considered of great interest to determine if alterations in β_1_-AR signal pathway occur in different types of cardiac diseases. It may be noted that pressure overload in cardiovascular diseases such as hypertension, aortic stenosis, and aortic valve stenosis is associated with an increase in the ventricular wall thickness (concentric cardiac hypertrophy). On the other hand, volume overload in pathological conditions such as anemia, heart block, regurgitant mitral or aortic valves, as well as atrial or ventricular septal defects, and different congenital diseases, is associated with dilatation of the left ventricle chamber (eccentric cardiac hypertrophy) [61, 158, 159]. Varying degrees of changes in β_1_-AR signaling system due to both pressure overload [160, 161, 162, 163, 164] and volume overload [165, 166, 167, 168, 169] have been observed at the end-stage heart failure. Alterations in β_1_-AR signal transduction have also been reported to occur in other types of heart diseases [170, 171, 172] and heart failure due to chronic myocardial infarction [173, 174, 175].

Downregulation of β_1_-AR has been shown to occur in patients with left heart valvular disease as well as chronic mitral regurgitation [166, 176]. Depressions in myocardial β_1_-AR density, adenylyl cyclase activity, and response to isoproterenol were observed after inducing volume overload [177]. A reduction in the adenylyl cyclase response to norepinephrine has been reported due to volume overload [167]. Furthermore, upregulation of β_1_-AR mechanisms was seen in the hypertrophic stage whereas these changes were depressed in heart failure [178]. Alterations in β_1_-AR signaling system, sensitivity of the myocardium to β_1_-AR stimulation, as well as changes in the subcellular distribution of regulatory proteins namely G-protein-coupled receptor kinase (GRK) isoforms and β-arrestins were observed at different stages of heart failure due to volume overload [165, 168]. Other studies have also shown increased β_1_-AR expression and GRK activity as well as depressed activities of different components of β_1_-AR signaling pathways in heart failure [169, 179, 180, 181]. Such variable alterations in β_1_-AR signal transduction system in the hypertrophied and failing hearts due to volume overload appear to be related to the stage of heart disease.

Varying degrees of changes in β_1_-AR, adenylyl cyclase and Gs-protein have also been identified in cardiac hypertrophy under several conditions associated with pressure overload [160]. Modification of cardiac adenylyl cyclase activities and changes in Gs-protein function have been observed in hypertension [172, 182]. Pressure overload induced heart failure in guinea pigs was accompanied by an increase in β_1_-AR density [183]whereas depressions in the density of β_1_-AR as well as isoproterenol-induced increase in cardiac contraction and stimulation of adenylyl cyclase activity were observed in dogs with heart failure due to pressure overload [161, 184]. Overexpression of cyclic AMP-hydrolyzing protein phosphodiesterase 4B (PDE4B), a key negative regulator of cardiac β_1_-AR stimulation, was shown to blunt the β_1_-AR signaling whereas its deficiency resulted in abnormal ${\mathrm{Ca}}^{2+}$-handling in pressure overload induced cardiac hypertrophy [185]. Furthermore, overexpression of a dominant negative mutant of Gsα-proteins decreased β_1_-AR responsiveness and protected against isoproterenol-induced cardiac hypertrophy in transgenic Gsα-DN-mice [186]. These observations showing variable changes in β_1_-AR signaling transduction system due to pressure overload also support the view that alterations in β_1_-AR signaling are dependent upon the stage of cardiac hypertrophy and heart failure.

## 5. Experimental Evidence for Alterations in β_1_-AR Mechanisms in Cardiac Hypertrophy

**Fig. 2.**
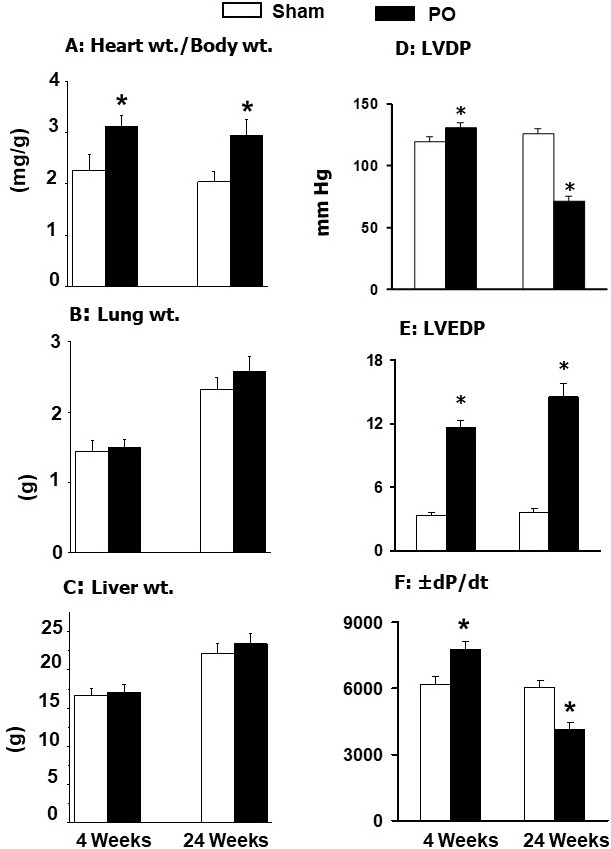
**General characteristics and ventricular function in rats at 4 and 24 weeks due to pressure overload (PO) after occluding the abdominal aorta**. Data are based on the results described in our paper —Journal of Applied Physiology. 2007; 102: 978–984 [42]. LVDP, left ventricle developed pressure; LVEDP, left ventricle end diastolic pressure; ± dP/dt, rates of rise and decline of ventricle pressures. *p < 0.05 versus respective sham.

Since heart failure is commonly associated with cardiac hypertrophy, we have evaluated the existing information to determine if alterations in β_1_-AR mediated activities in the failing hearts are a consequence of the hypertrophic process. In this regard, we monitored changes in β_1_-AR signal transduction in pressure overload induced cardiac hypertrophy which was induced upon occluding the abdominal aorta in rats for 4 and 24 weeks [34, 42, 172, 187, 188]. The results in Fig. 2 (Ref. [42]) indicate that increased heart weight/body weight ratio (an index of cardiac hypertrophy) at 4 weeks of pressure overload was accompanied by increased left ventricle developed pressure (LVDP), left ventricle end-diastolic pressure (LVEDP) as well as rates of both rise and decline of ventricular pressures (± dP/dt) without any changes in the lung or liver weight to body weight ratios. On the other hand, hypertrophy induced by pressure overload for 24 weeks was associated with increased LVEDP and depressions in both LVDP and ±dP/dt parameters without any changes in lung or liver weight to body weight ratios (Fig. 2). These observations suggest that pressure overload for 4 weeks induces adaptive hypertrophy whereas that for 24 weeks induces maladaptive hypertrophy without any changes in lung or liver congestion (well-known indices of heart failure).

**Fig. 3.**
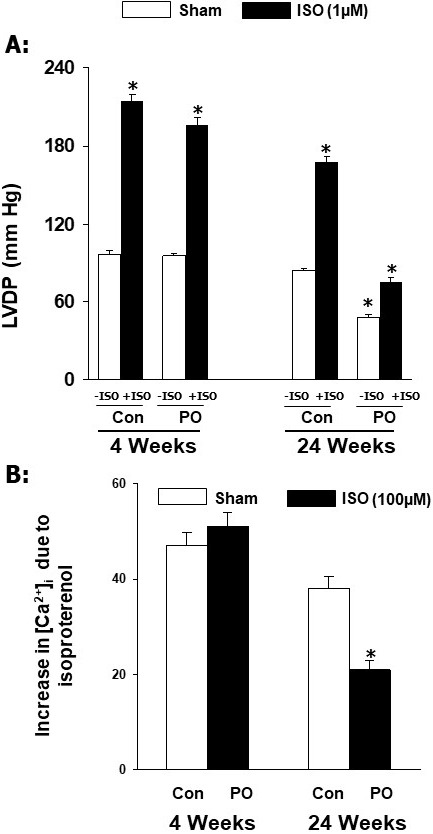
**Effects of isoproterenol (ISO) on ventricular developed pressure and [${\mathrm{Ca}}^{2+}$]_𝐢_ in cardiomyocytes at 4 and 24 weeks due to pressure overload (PO) in rats**. Data are based on the results described in our paper —Journal of Applied Physiology. 2007; 102: 978–984 [42]. Con, control; LVDP, left ventricle developed pressure. *p < 0.05 versus respective sham.

**Fig. 4.**
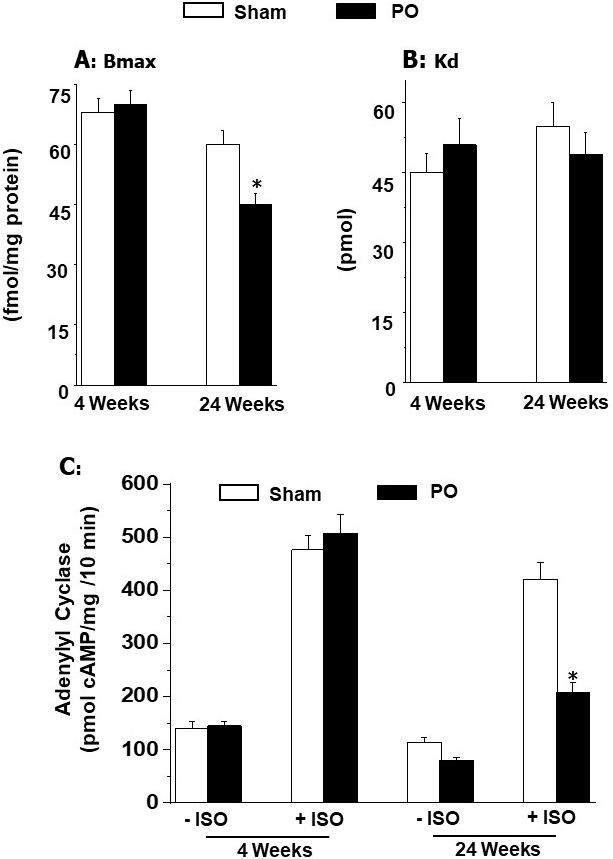
**Ventricular $B_{\text{𝐦𝐚𝐱}}$ (maximal number of binding) and $K_{\text{𝐝}}$ (dissociation constant) values for β_1_-adrenoceptors and effect of isoproterenol (ISO) on adenylyl cyclase activity at 4 and 24 weeks due to pressure overload (PO) in rats**. Data are based on the results described in our paper — Journal of Applied Physiology. 2007; 102: 978–984 [42]. *p < 0.05 versus respective sham.

Fig. 3 (Ref. [42]) shows that increased cardiac function (as reflected by increase in LVDP) and intracellular ${\mathrm{Ca}}^{2+}$-concentration ([${\mathrm{Ca}}^{2+}$]_i_) in cardiomyocytes by isoproterenol were not affected in adaptive hypertrophy due to pressure overload at 4 weeks. In contrast, both isoproterenol-induced increase in LVDP in the heart and [${\mathrm{Ca}}^{2+}$]_i_ in cardiomyocytes were depressed in maladaptive hypertrophy due to pressure overload at 24 weeks. Furthermore, the results in Fig. 4 (Ref. [42]) show that pressure overload induced adaptive hypertrophy for 4 weeks did not show any changes in β_1_-AR density ($B_{\text{max}}$ value); without any changes in dissociation constant ($K_{\text{d}}$ value) or isoproterenol-induced increase in adenylyl cyclase activity. In contrast, pressure overload reduced maladaptive hypertrophy for 24 weeks showed depressions in β_1_-AR density and isoproterenol-induced increase the adenylyl cyclase activity (without any changes in $K_{\text{d}}$ value) (Fig. 4). These data have been interpreted to reflect that adaptive cardiac hypertrophy due to pressure overload did not show any changes in β_1_-AR signal transduction mechanisms whereas maladaptive cardiac hypertrophy due to pressure overload was associated with some defects in the β_1_-AR signaling.

## 6. Experimental Evidence for Alterations in β_1_-AR Mechanisms in Heart Failure

**Fig. 5.**
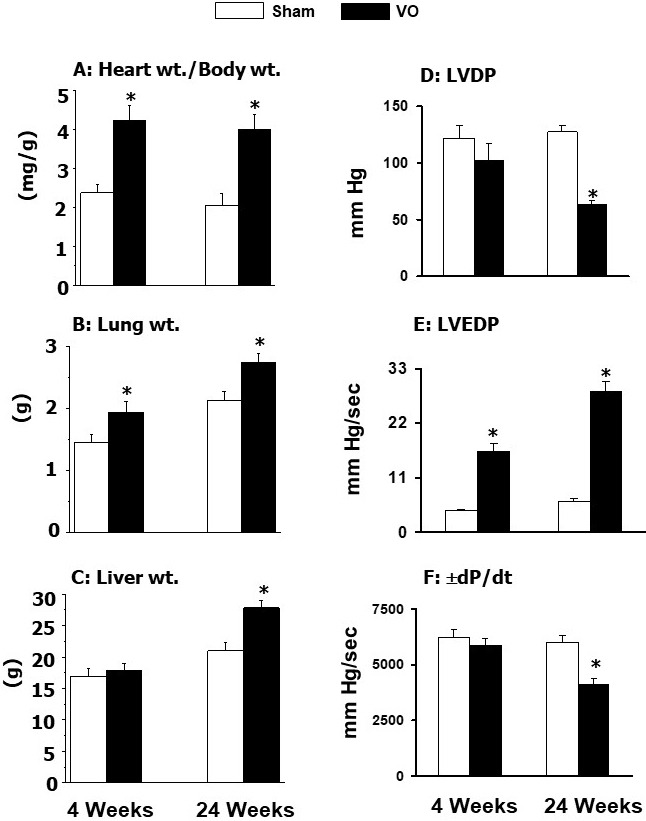
**General characteristics and ventricular function in rats at 4 and 24 weeks due to volume overload (VO) after the aortocaval shunt**. Data are based on the results described in our paper — Journal of Applied Physiology. 2007; 102: 978–984 [42]. LVDP, left ventricle developed pressure; LVEDP, left ventricle end diastolic pressure; ± dP/dt, rates of rise and decline of ventricle pressure. *p < 0.05 versus respective sham.

In order to show if changes in β_1_-AR signal transduction system in heart failure are similar to those seen in adaptive cardiac hypertrophy, the data from studies in which volume overload was induced by aorto-venous (AV) shunt in rats at 4 and 24 weeks was evaluated [42, 84, 165, 168, 169, 189, 190]. The results in Fig. 5 (Ref. [42]) show that increased heart weight to body weight ratio was accompanied by increased LVEDP and lung weight to body weight ratio without any changes in LVDP, ±dP/dt and liver weight to body weight ratios upon inducing AV-shunt for a 4-week period. It is pointed out that since no changes in cardiac function (as represented by LVDP and ±dP/dt parameters) were evident upon inducing volume overload for 4 weeks, we believe that cardiac hypertrophy at this stage is of adaptive type. Since lung weight to body weight ratios was significantly increased at 4 weeks of inducing volume overload, it can be argued that it may represent an early stage of heart failure. However, this may not be the case as no changes in cardiac function were observed at this stage. On the other hand, increases in heart weight to body weight ratio and LVEDP upon inducing volume overload for 24 weeks were associated with depressions of both LVDP and ±dP/dt as well as increases in both lung or liver weight to body weight ratios, indicating the occurrence of heart failure. These data are consistent with the view that adaptive cardiac hypertrophy and heart failure due to volume overload become evident at 4 weeks and 24 weeks after inducing AV-shunt, respectively.

**Fig. 6.**
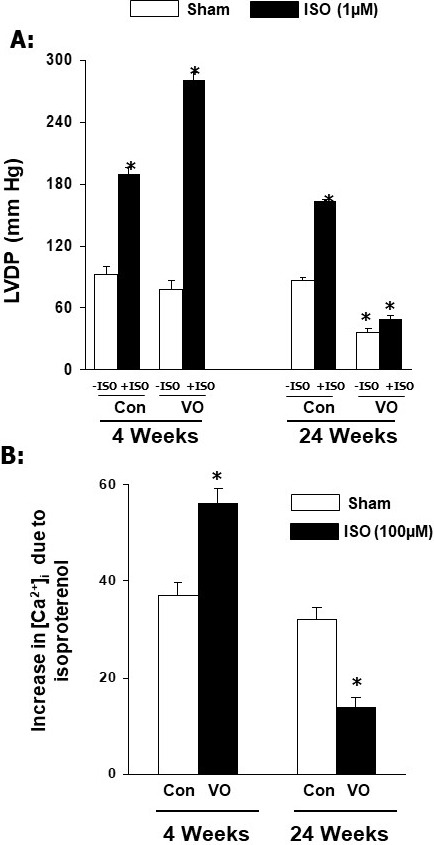
**Effects of isoproterenol (ISO) on left ventricular developed pressure (LVDP) in rats and [${\mathrm{Ca}}^{2+}$]_𝐢_ in cardiomyocytes at 4 and 24 weeks due to volume overload in rats**. Data are based on the results described in our paper —Journal of Applied Physiology. 2007; 102: 978–984 [42]. LVDP, left ventricle developed pressure; Con, control; VO, volume overload. *p < 0.05 versus respective sham.

**Fig. 7.**
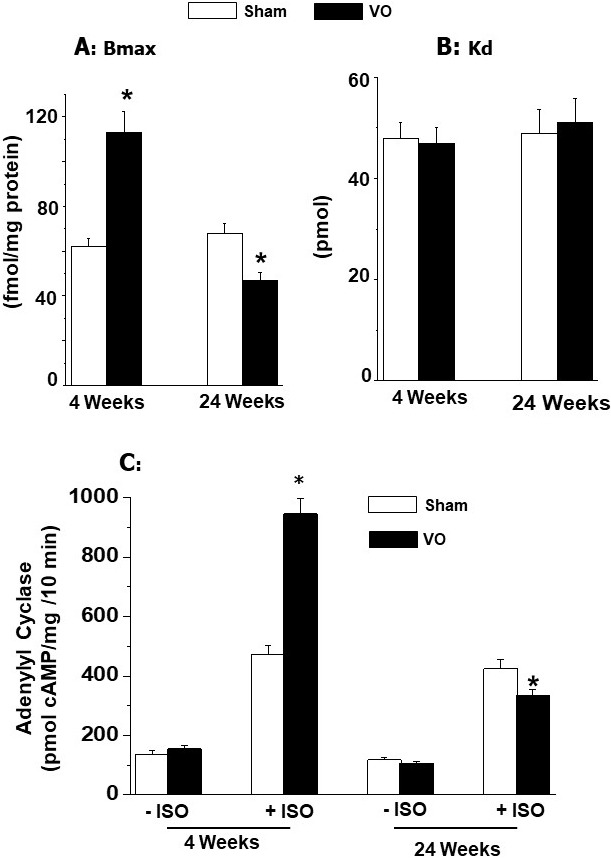
**Ventricular Bmax (maximal number of binding) and Kd (dissociation constant) values for β_1_-adrenoceptors and effect of isoproterenol (ISO) on adenylyl cyclase activity at 4 and 24 weeks due to volume overload (VO) in rats**. Data are based on the results described in our paper —Journal of Applied Physiology. 2007; 102:978–984 [42]. *p < 0.05 versus respective sham.

The results described in Fig. 6 (Ref. [42]) indicate that isoproterenol-induced increases in LVDP in the heart and [${\mathrm{Ca}}^{2+}$]_i_ in cardiomyocytes were augmented by volume overload at 4 weeks of inducing AV-shunt whereas these responses of the heart to isoproterenol showed marked depressions at 24 weeks AV-shunt. Furthermore, β_1_-AR density as well as activation of adenylyl cyclase by isoproterenol were markedly augmented by volume overload at 4 weeks after inducing AV-shunt whereas both β_1_-AR density and isoproterenol-induced activation of adenylyl cyclase were attenuated at 24 weeks after inducing AV-shunt. No changes in $K_{\text{d}}$ values for β_1_-AR were observed either at 4 weeks or 24 weeks after inducing AV-shunt (Fig. 7, Ref. [42]). These data indicate that alterations in β_1_-AR signal transduction pathways in the failing heart are not similar to those in adaptive cardiac hypertrophy due to volume overload.

## 7. Conclusions and Perspectives

Although heart failure is associated with cardiac dysfunction, there also occurs a loss of adrenergic support, which is considered to maintain cardiac performance in this syndrome. The depression of inotropic responses to stimulation of the SNS or exogenously administrated catecholamines is considered to be a consequence of a defect in the β_1_-AR signal transduction in heart failure. However, the exact mechanisms for such an alteration are not fully understood. Since the β_1_-AR signaling system is known to include β_1_-AR, Gs-and Gi-proteins and adenylyl cyclase, it has been observed that alterations in anyone of these components may result in reduced formation of cyclic AMP and subsequent impaired PKA-mediated phosphorylation of subcellular proteins in the failing heart. In view of the importance of β_1_-AR signaling and PKA-induced phosphorylation of ${\mathrm{Ca}}^{2+}$- pump and ${\mathrm{Ca}}^{2+}$- release proteins in the sarcoplasmic reticulum as well as troponin and other regulatory proteins in myofilaments for regulating cardiac function, it is likely that augmentation and depression of isoproterenol - induced responses of cardiac function in adaptive cardiac hypertrophy and failing hearts are due to corresponding alterations in PKA associated phosphorylations [13, 14, 32, 34, 63, 65], respectively. In fact, various studies in heart failure have shown that the depressed β_1_-AR signaling in failing hearts is due to desensitization of β_1_-AR [67, 74, 85] but these changes are considered to be dependent on the stage of heart failure. Since catecholamines for a short period increase cardiac contractile force whereas these responses are attenuated over a prolonged period, it appears that downregulation of β_1_-AR signal transduction in heart failure may be due to elevated levels of plasma catecholamines for a prolonged period. It is also pointed out that oxidative stress plays an important role in the pathogenesis of heart failure and it is likely that defects in β_1_-AR signaling at the advanced stage of heart failure may be due to the development of oxidative stress as a consequence of circulating catecholamines and other vasoactive hormones such as angiotensin II [34, 80, 191]. Accordingly, it is suggested that therapy of heart failure with some antioxidants may prove useful in preventing downregulation of β_1_-AR mechanisms in the failing heart.

From the foregoing discussion, it is evident that not only changes in β_1_-AR signal transduction are dependent upon the stage of heart failure, marked differences in β_1_-AR signaling have also been observed in adaptive and maladaptive cardiac hypertrophy. Particularly, it is noteworthy that adaptive hypertrophy induced by pressure overload or volume overload for a 4-week period was found to exhibit either unaltered or augmented responses of heart function, [${\mathrm{Ca}}^{2+}$]_i_ in cardiomyocytes and adenylyl cyclase activity to isoproterenol as well as unaltered or increased β_1_-AR density. On the other hand, all these responses or parameters for β_1_-AR signal transduction mechanisms were depressed in maladaptive hypertrophy at 24 weeks of inducing pressure overload as well as in heart failure at 24 weeks of inducing volume overload. Such differences in β_1_-AR signaling in adaptive and maladaptive cardiac hypertrophy as well as heart failure can be explained on the basis of differences in the development of progressive levels of oxidative stress as a consequence of circulating catecholamines and other vasoactive hormones for a prolonged duration [143, 191]. Furthermore, it is pointed out that, unlike the adaptive cardiac hypertrophy, both maladaptive cardiac hypertrophy at 24 weeks due to pressure overload and heart failure due to volume overload for 24 weeks were found to exhibit a similar pattern of depressions in all parameters of β_1_-AR signal transduction system. Thus, it appears that downregulation of the β_1_-AR signaling in heart failure or maladaptive cardiac hypertrophy may not be associated with the hypertrophic process *per se*. Although occurrence of oxidative stress has been suggested to be involved in transition of adaptive hypertrophy to maladaptive hypertrophy as well as progression to heart failure [80, 143, 191], extensive research work needs to be carried out with respect to establishing any relationship between oxidative stress and changes in β_1_-AR signal transduction pathway during the development of heart failure to make any meaningful conclusion.

Several investigators have reported a wide variety of changes in β_1_-AR signal transduction in cardiac hypertrophy and heart failure [25, 34, 35, 46, 54, 73, 149]; however, the exact mechanisms for such variable alterations in this pathway have not been identified. It needs to be emphasized that adaptive cardiac hypertrophy has been suggested to be a consequence of changes in the redox status of myocardium due to formation of a small amount of oxyradicals [137, 191]. On the other hand, excessive formation of oxyradicals for the occurrence of oxidative stress is considered to be involved in the development of maladaptive cardiac hypertrophy and subsequent heart failure [137, 191]. However, the participation of other mechanisms such as alterations in the levels of proinflammatory cytokines and intracellular ${\mathrm{Ca}}^{2+}$ - overload as well as metabolic abnormalities [51, 60, 61, 136, 138, 155, 192] cannot be ruled out for explaining the difference in the status of β_1_-AR signaling in non failing and failing hypertrophied hearts. Since the activation of baroreceptors in the heart is known to play a critical role in the regulation of cardiac function and β_1_-AR mechanism [193], alterations in the baroreflex mechanisms during the development of hypertension and heart failure have been implicated in changing the intensity of adrenergic stimuli and β_1_-AR signal transduction pathway [194, 195]. This view is also supported by the observations that there occurs an increase in the sympathetic activity and a decrease in the parasympathetic activity in patients with heart failure [196]. In addition, newer approaches for activating the baroreflex system or vagal stimulation have been shown to exert promising effects in correcting the autonomic imbalance for improving cardiac performance in heart failure [197, 198]. Accordingly, progressive changes in the baroreflex system due to both pressure and volume overload can also be seen to induce upregulation and downregulation of β_1_-AR signaling during the development of cardiac hypertrophy and heart failure. Thus, it appears that the pathophysiological and molecular mechanisms in changing the status of β_1_-AR signal transduction pathway in cardiac hypertrophy and heart failure are of complex nature and require further studies for establishing the exact relationship among diverse pathogenic factors for the induction of alterations in β_1_-AR signaling.
